# *Spare Rib*, The British Women’s Health Movement and the Empowerment of Misery

**DOI:** 10.1093/shm/hkab016

**Published:** 2021-07-23

**Authors:** Zoe Strimpel

**Keywords:** health, women’s liberation movement, women’s health movement, spare rib, feminism, feminist periodicals, bodies, embodiment, sexual health

## Abstract

This article elucidates *Spare Rib’*s (1972–1993) value as a source for considering the UK women’s health movement of the 1970s and 1980s. It focusses on the magazine’s role in mediating and shaping its readers’ relationship to negative bodily experience, and outlines its distinctiveness within the broader feminist landscape of women’s health coverage. While aspects of the British women’s health movement have attracted scholarly attention, such as mental health activism and abortion campaigns, we still know relatively little about how non-activist British women interpreted their bodily experiences and health through the feminist lens offered from the early 1970s. The first part of the article focusses on *Spare Rib*’s contribution to the British women’s health movement. The second part of the article zooms in on a selection of letters that show how readers took up, and found empowerment, in modes of bodily disclosure fostered by the magazine.

‘I know no woman—virgin, mother, lesbian, married, celibate—whether she earns her keep as a housewife, a cocktail waitress, or a scanner of brain waves—for whom her body is not a fundamental problem: its clouded meaning, its fertility, its desire, its so-called frigidity, its bloody speech, its silences, its changes and mutilations, its rapes and ripenings … There is for the first time today a possibility of converting our physicality into both knowledge and power’ –                     Adrienne Rich, *Of Woman Born*, p.284^1^

This article elucidates *Spare Rib’*s (1972–93) value as a source for considering the UK women’s health movement of the 1970s and 1980s. It focusses on the magazine’s role in mediating and shaping its readers’ relationship to negative bodily experience, and outlines its distinctiveness within the broader feminist landscape of women’s health coverage. Health was a foundational issue of the Women’s Liberation Movement, and a literal site for realising the political nature of the personal in which women could pivot between accounts of personal experience (bodily discomfort, shame, sense of freakishness) and political critiques of patienthood in the hands of state-run medicine (sexist doctors, patriarchal health agendas around contraception and reproductive health, unfair distribution of economic and social resources that took a toll on women’s health).^2^ Yet while aspects of the British women’s health movement have attracted scholarly attention, such as mental health activism and abortion campaigns, we still know relatively little about how apparently non-activist British women interpreted their bodily experiences and health through the feminist lens offered from the early 1970s.^3^*Spare Rib*, the UK’s only national feminist magazine, functioned as the movement’s broadest and most sustained exchange between activists and women of different degrees of feminist involvement. It was a conduit for information and experience between a wide range of activist and non-activist women, readers, writers, theorists and specialists around the UK and the world. It was therefore a key source for considering how health was appropriated and presented by the Women’s Liberation Movement to a wider audience of UK women. *Spare Rib* readers were not a general audience; they were at the very least interested or intrigued by feminism. But unlike more hard-boiled feminist publications such as *Shrew* and *Red Rag*, the magazine ‘felt the need to persuade women to the cause’ and so strove, especially in its first years, to appeal to as wide a readership as possible with ‘varied layout, tone and types of feminism’.^4^ The letters focussed on here come from women struggling with some aspect of their health, but whose tone and content do not suggest a background or history in activism; rather, *Spare Rib* appears to be the nexus of their relationship to the feminist movement (though this does not rule out activism).

I begin by offering an account of *Spare Rib* in the context of the British women’s health movement, exploring why it was unique, particularly in the kind of reader disclosures it invited. The second section is concerned with how women responded to the magazine’s health coverage and articulated their own relationship to health and their bodies in the correspondence page. This section is divided into two chronological periods to reflect first the magazine’s central period, from founding to the late 1980s. A concern with all aspects women’s health crested and remained strong in this period, reflected in a vigorous reader discourse in the letters pages. The second period concerns the last years of *Spare Rib*, caught up in internal disagreement and instability in which concerns around race and new organisational emphases substantially reconfigured the magazine, fracturing the readership and inflaming disagreements over the place of personal testimony in the magazine. The focus in this section is on a selection of letters that show how readers took up, and found empowerment, in modes of bodily disclosure fostered by *Spare Rib*. Articulations of ill health were fundamental not only to the political aims of the magazine but also the development of readers’ own political understandings. In being encouraged to offer such detail, readers were given distinctive opportunities for articulating their sense of belonging and connection to women’s liberation. In addition, their letters offer a glimpse into the infra-institutional, everyday vernaculars of women’s health circulating in and around the women’s liberation movement.

Periodical communities like *Spare Rib’*s belonged to a rich ecosystem both of consciousness raising (CR) and health activism. But print, with anonymity offered on request, offered different, not fewer, dynamics and opportunities for women’s self-expression as CR groups, and offered a set of heterogeneous, open-ended possibilities for self-narration not necessarily on offer at health groups, which tended to be convened around a particular issue or to a particular end.^5^ Readers could write freely, controlling or experimenting with their expression, without the pressure of immediate social response. The liberatory promise of the epistolary form in relation to bodily trouble or confusion had been clear since earlier in the century, for instance in the barrage of frank and desperate letters to Marie Stopes after the publication of *Married Love* in 1918. Letter writers to Stopes, many of whom were men, requested information and were not encouraged to explore a political stance on their own sexuality.^6^ Moreover, as Lesley Hall has shown in her study of mid-century male sexual anxieties, much of this correspondence was inscribed with a sense of powerlessness and supplication.^7^ The Women’s Liberation Movement, by contrast, introduced a distinctive epistolary culture rooted in new, anti-hierarchical modes of transparency and self-exploration. As Margaretta Jolly has shown, letters between feminists have been key means for fusing emotional and political worlds and provided a means by which women both developed their sense of feminist identity and articulated their affective expectations of each other.^8^ In circulating between strangers, *Spare Rib* letters offered a mechanism of self-realisation oriented towards political rather than intimate ends, in which readers could develop confidence in thinking, writing and acting in relation to the body, and, from there, society more broadly.

This article is in dialogue with scholarship showing how strong feelings of anger and misery have sustained and enabled the emotional logics of feminist periodicals. In her article on the affective networks of suffrage periodicals, Green has noted that the expression of ‘melancholic feelings of alienation and isolation [in *Votes for Women*] become the ties that bind individuals to a common experience’.^9^ Building on Green’s analysis for the 1970s period, and closer to the terrain of this article, Melanie Waters has discussed the affective opportunities and dynamics of anger in *Spare Rib’*s correspondence pages, deploying Lauren Berlant’s notion of an ‘intimate public’ to read the strong, often negative, emotions on the pages.^10^ Yet the focus of both Green and Waters is on how reader feeling was ‘usefully instrumentalized in service to feminism’s political ends’.^11^ This article switches the focus, investigating how *Spare Rib* was used in service to individual *readers’* political ends, rather than vice versa. It suggests that the letters to the magazine relating to bodily experience offer an especially clear lens for viewing the process by which readers rendered the personal political.

## The Feminist Body: The British Women’s Health Movement and *Spare Rib*

The British Women’s Health movement of the late twentieth century has been divided into two main phases. According to Lesley Doyal’s influential analysis, the 1970s was a period of ‘growing awareness that certain areas of knowledge that had previously been monopolised by doctors were potentially of immense value to women—knowledge about how our bodies work, for example’.^12^ Informational networks, such as women’s health groups and their newsletters, flourished alongside CR groups, the distinctive means by which the Women’s Liberation Movement facilitated political self-empowerment and awareness.^13^ The second phase emerged in the decade after the Conservatives came to power in 1979 presenting urgent challenges to state-run healthcare.^14^ Feminist health activism saw a shift in emphasis towards ‘the broadening of the basis of political activity to include a concern with the defence of the NHS’ forging a ‘much stronger link between women as providers and women as users of health care’.^15^ The link was strengthened through a proliferation of clinics, centres and other women’s health services offering screening, tests and advice, as well as activist trade groups, newsletters and magazines representing midwives, nurses, pregnancy testers, and other women health workers.^16^

The growing organisational reach of the British women’s health movement after 1970 can be seen in the expansion of Well Woman Clinics, a nationwide network that came to be situated within an NHS framework, offering screening and information.^17^ The first Well Woman clinics emerged out of CR culture. The Liverpool Road clinic, first held in 1973, was formed by members of the Essex Road Women’s Health Group and prioritised sharing experience and offering support in a group setting, along with expert advice.^18^ In 1975, 531 women came to two clinics; in 1980 it was 1569 to 5 clinics, in step with nationwide expansion.^19^ Despite becoming more mainstream, Well Woman clinics kept their feminist framework in place. An appendix on a 1979 Well Woman scoping report for a Manchester Clinic included a comment by the Manchester WLM saying it fully supported the provision of Well Women Clinics but was concerned that they resist heteronormativity.^20^ It suggested that lesbians and celibate women should not be excluded and was displeased at the idea that abortion referral would be excluded. However by the 1990s, Well Woman clinics had come to signal a new era and paradigm for thinking about women and health in a mainstream way, with a pragmatic approach that kept politics to a minimum—in any case most women who attended the clinics were there for blood tests and screenings and to avoid ‘being barraged by yes-or-no questions about your medical past’.^21^

Uniting the women’s health movement across discursive and clinical spaces was an insistence on the need for an epistemological overhaul in which ‘objective’ clinical knowledge should no longer be seen as superior to women’s subjective knowledge of their own bodies.^22^ Reaching across the decades after its initial publication and now available in 31 languages, the American text of *Our Bodies Ourselves* (*OBOS*) had provided the early transformative framework for this shift.^23^*OBOS* attained global reach and was translated and published widely around the world, but emerged from the meetings of the Boston Women’s Health Book Collective, forged from women’s workshops in the late 1960s. The book evolved through a process of information gathering, experience and discussion papers, and an ethos that ‘validated women’s embodied experiences as a resource for challenging medical dogmas …. and, consequently, as a strategy for personal and collective empowerment’.^24^ Accessible and warm, the first *OBOS* manual (1970) was an immediate success, with unexpected offers from commercial publishers soon following, and leading to a commercial edition in 1973, which would go on to sell millions of copies. In 1978—following Japanese, Italian, Danish and French editions—the first British edition of *Our Bodies Ourselves*, edited by two *Spare Rib* health writers, Jill Rakkusen and Angela Phillips, was published, adapted for the distinctive landscape of the NHS.^25^*OBOS* enshrined a feminist form of expertise: self-educated women, including those without formal qualifications delivering dynamic reams of demystifying information to a wide range of readers, while delivering tangible impact on health policy.^26^ Its format precluded the ricocheting, accruing nature of expertise between readers possible in a letters section like *Spare Rib’*s, but in performing a ‘labour of language’, it had opened new modes of frank and probing self-expression.

The women’s liberation movement’s success in carving out both new subjective languages of embodiment and radically detailed medical explanations should be seen in relation to long-standing, deeply embedded cultures of mystery, shame and misinformation about women’s bodies and particularly their reproductive organs.^27^ As Lesley Hall and Roy Porter have discussed through the lens of sex advice literature, by the Victorian era the study of female anatomy, when linked to sexuality and desire, was so contentious that, in some cases, it was dismissed as beyond the pale for inclusion in publicly available science, even by respectable medical men.^28^ Medical knowledge had been formalised and disseminated by men.^29^ Legally, women could enter the medical profession from 1876, but medicine remained male dominated throughout the twentieth century. The science of sexology expanded in the Edwardian period, changing codes of expression and knowledge around female embodiment. Yet as Hera Cook has shown, the premium increasingly put on ‘hygiene’ at the same time highlighted just how poor women’s bodily knowledge remained—many girls ‘confused the pleasurable mess of sex with the dirt of excreta’.^30^ And although women began to enter and indeed drive new discourses of bodily knowledge in relation to birth control, the agenda remained conservative—focussed on sex among the married—rather than frank and exploratory for all.^31^ Despite enlargement of public discourse surrounding aspects of female sexuality from the 1920s, women’s relationship to their bodies remained highly mediated by male-dominated institutions into the late twentieth century. Medical schools retained sex quotas into the 1970s and only one in seven doctors were female, while gynaecology remained male dominated in the decade.^32^ The task facing the Women’s Movement’s health writers and activists was clear: as late as 1978, in health writer Nancy Mackeith’s view, close self-examination of genital problems by women was seen ‘by many people’ as a sexual perversion and an obscene interest in ‘private parts … To some people a woman knowing her cervix and her vagina is too powerful a tool for her to cope with … she might get an infection … [become] dangerously frightened … [and threaten] her relationship with the medical profession’.^33^ Meanwhile new discursive regimes of bodily containment intensified in the late twentieth century, linked to what feminists saw as a toxic fusion of Thatcherite individualism and neoliberalism. In 1989, writing in the introduction to the updated edition of British *OBOS*, former *Spare Rib* writers Jill Rakusen and Angela Philips reflected on the ‘healthism’ of the ‘economic and political climate’ of the 1970s and 1980s.^34^ As Rakusen and Philips pointed out, in mainstream magazines and media, as well as institutional therapeutic narratives, women were told that wellbeing lay in mastering the body, and that solutions to being overweight, depressed, lethargic or infection-prone lay in a commitment to hard self-work and disciplining. Commercial imperatives to female bodily containment were ubiquitous: by the 1970s, the brutal hygiene regimens advised by doctors in the 1920s had become less medicalised and more commercialised.^35^ Vaginal deodorants saw a surge in marketing and popularity. Femfresh, Bidex and Fresehette spent £37,000 each on print ads and by the 1970s 15 per cent of women were using vaginal deodroants—this rose to 30 per cent in the 16–24 group.^36^ When it came to advertising for feminine and beauty products, or adverts with sexist messages, even *Nova* (1965–1975), the ‘politically radical … intellectual women’s magazine’ with clear feminist alignments and a critical eye on prevailing gender norms, showed itself to be ethically unopposed.^37^ It ran adverts from vaginal deodorant Freshette (‘No woman is perfect’) and Wella (‘hair colour you can use at home—with confidence’) as well as a host of other clients that put pressure on women to be thin, contained, pretty and sexy, from British Airways (‘It takes a special kind of girl [stewardess] to inspire this kind of trust’) to Crème de Menthe (‘only drink it if you believe a good book is enough work for one day’ accompanying a sultry young and toned woman reclining in a hammock).^38^

Meanwhile, the pressures on women to control and reduce their bodies led to soaring rates of diagnoses of anorexia and bulimia in the 1970s, as well as issues related to weight gain, both of which became areas of feminist interest.^39^ Sam Baker, former editor of *Cosmopolitan*, *Red* and *Company* magazines discussed in a recent oral history the complex editorial obligations that came with a large advertising base among beauty clients, noting that women’s magazines could neither be explicitly ‘political’, nor review any of their advertisers’ products negatively.^40^ In being free of the need to please advertisers of beauty and hygiene products, *Spare Rib’*s health coverage could operate against the grain of mainstream beauty and health markets encouraging women to contain, cleanse, smooth and make aromatic their bodies.^41^

## *Spare Rib* as Distinct

Like other health-related writing (newsletters, pamphlets) circulating the movement, *Spare Rib* offered a brisk tone of detail-packed health coverage. A 1978 feature on hirsutism was typical, in response to an article in *Woman’s Own* that summer that had linked hirsutism to careerism. ‘We’ve learnt a lot about the way our bodies work’, it began, but body hair was still a taboo, mysterious and shameful topic.^42^ There was a box on hirsutism’s organic causes, plus a short history of depilation over the ages, reminding readers that mainstream notions of femininity were obfuscatory and damaging when it came to physical disorders of excess or lack.

Such an approach created a welcoming environment for readers’ detailed personal disclosures, though *Spare Rib* was not the only feminist publication that printed harrowing or detailed first-person accounts of bodily misery. The health movement’s growing network of groups, newsletters, questionnaires and research documents fostered a range of opportunities for self-narration. However, first person accounts tended to relate to the single issue the publication was concerned with, such as childbirth letters in the Association for Improvements of Maternity Services journal or the NCT newsletter.^43^ And such accounts tended to be marshalled by the researcher, the author, or the editor around a particular theme, with first-person testimony kept brief (and jostling for space with the informational agenda of the author, editorial collective or researcher). Extensive, regular periodical space for the testimony of female patients was not visible in a national platform. And *Spare Rib* was a particularly broad site of feminist exchange, inviting substantive contributions from readers across sections. It invited and depended on contributions from individual women in letters, first-person features, columns such as Tooth and Nail, where readers sent in examples of the most egregious sexist advertising, and Odds and Sods (individual requests for advice or information, or complaints) as well as Classifieds.^44^ It therefore brought testimonies from a wide range of encounters across women’s health services and standard NHS provision. *Spare Rib’*s letters pages could afford to be experimental: women could vent, explain, posit theories and solutions and invoke politics. Women’s health groups and centres’ resources, by contrast, went into offering practical support, and acting as a mediator between women and local authority and state-run health.^45^ The 1979 scoping report on Well Woman in Manchester proposed ‘Adequate time for women to discuss their problems … to find out the exact nature of the problem … ‘.^46^*Spare Rib* offered something different: it was a space to express disempowerment and explicitly unempowered body experience.

*Spare Rib* was also distinct in the kinds of first-person testimonies it elicited from readers about their bodies. Other women’s magazines took women’s health seriously and ran letters from women on a range of issues relating to physical and mental health, but their emotional and epistemological scope was different. To illuminate the uniqueness of *Spare Rib’*s epistolary offering, it is worth briefly considering three competitors to *Spare Rib*: *Nova, Cosmopolitan and Company*, each of which either advanced a highly mediated version of feminism and engaged with ideas aligned with the women’s liberation movement frequently if ambivalently and often critically.^47^ Even in the relatively progressive *Nova* (1965–75), the closest commercial offering to *Spare Rib*, a man was given space to intervene on the precise nature and style of female bodily disclosure.^48^ ‘I was irritated to read another pointless and petulant article on women’s bodies [by Bel Mooney in August 1974 Nova],’ wrote Graham Norman of Leicestershire, ‘in which she disregards the obvious fact that millions of women really appreciate the benefits of bras, tampons, deodorants and perfumes, and have also, without lengthy soul-searching, come to terms with the functions of their own bodies … ’.^49^ The first issues of *Spare Rib* included letters from men, but a letter like this is hard to envisage at any time.

Most *Nova* letters came from women, of course, and while some of these made links with structural inequalities, they rarely led with or even saw the reader’s experience in gender-political terms. Also in the January 1975 edition, a woman wrote a letter about post-natal depression, noting insufficient provision for it but shying away from feminist analysis and noting that ‘A similar pattern of events may occur for men’. Three letters on episiotomy included the observation by one reader that ‘doctors are deliberately steering all pregnant women into hospital’. This reader, however, attributed blame not to a sexist medical establishment but to the desire to make births ‘convenient for doctors and nurses’ in equal measure. Another writer accused *Nova* of making childbirth seem scary and therefore harder to do naturally. A short note from Sheila Kitzinger, the natural childbirth activist, requesting women’s experiences of episiotomy, concluded the triplet. *Nova’*s letters engaged frankly with the mis-handling, misinformation and under-funding surrounding women’s reproductive health problems, but readers were less preoccupied with the political nature of these issues than by the desire to find solutions. Experience was shared frankly, but rarely in the same negative emotive terms as those found in *Spare Rib*, and without the level forensic detail that *Spare Rib’*s letters so striking.

*Cosmopolitan*, like *Nova*, engaged directly (if ambivalently) with the women’s liberation movement, made explicit connections (sometimes negative) between single and sexually self-knowing womanhood and women’s liberation, and was more interested in sex and lifestyle than in health. It invited disclosures from readers, sometimes intimate, about sexual experience and outlook—but the tone, in keeping with the magazine’s branding, was one of empowerment and fun. ‘Then whoopee!’ wrote a reader living in Austria whose boyfriend posted her the magazine. ‘Cosmo comes through the post reminding me of that delicious world where you can do what you want and be what you want to be’.^50^ The realities of pain and suffering did not fit that ‘Cosmo’ model. Letters that did cover bodily misfortune often sought or offered practical solutions that prioritised the cosmetic over the political. A letter entitled ‘hair-erasing’ contrasted with *Spare Rib’*s coverage of hirsutism (discussed below): ‘I was shocked to see [the reply in June 1983 *Cosmopolitan*] to a lady who was worried about nipple hairs. Are you aiming to help women with their problems? If so, then why doesn’t the doctor tell the lady to have electrolysis? … in the long run it’s well worth having because it gets rid of the hairs *for good*.’^51^*Company* (launched 1977), another robustly cheerful lifestyle magazine aimed at empowering single women through straight-talking articles about life, work and love, ran about five letters per issue. Health occasionally attracted its own space for first-person accounts, but these were packaged in terms of honesty, rather than misery, pain or terrible diagnoses. In Loose Talk: You Answer Back (January 1989), readers’ response to a previous Loose Talk (October 1988) on ‘the whole business of being a woman’ were printed. Questions answered included: ‘Do you check your breasts regularly?’. ‘When did you last have a smear test?’ and ‘Do periods have an adverse effect on your life?’ Answers were from women who may have had sloppy self-examination practice, or unpleasant periods, but who were still healthy. Within this framework, as with *Nova*, there was some reference to sexist structures: ‘Male doctors only know the theory of the functions of a woman’s body—they can never begin to understand how you feel’, wrote one.^52^

*Spare Rib* offered women something different in terms of volume and space for correspondence as well as inflection. Running roughly twice as many letters as *Cosmopolitan* and *Company*, *Spare Rib* ran two to three pages of correspondence per issue, containing between seven and 16 letters which varied greatly in size, despite notices requesting writers to keep length down to aid their chances of inclusion. The collective’s selection methods for letters are not explicit, but as is clear from the *Spare Rib* collection at Feminist Archive South, the mailbag offered far more than could be printed. A host of harrowing letters detailing health tragedies and poor luck, and outrageous encounters with a sexist establishment, are to be found marked ‘not for pub’; it seems those letters which outlined their agenda most cogently, or which touched on problems that might not have been dealt with or aired in other fora were chosen. Neither shocking detail nor anger were grounds for exclusion, though, and it wasn’t until the letters following on from a sequence of editorials on Israel and Palestine that issues of censorship were raised by outraged readers who were used to, and expected, a correspondence section of extremes.

Until the final issues of the magazine, between two and four out of ten-16 letters concerned health in some way, whether that was working conditions for nurses or encomiums from midwives about the empowering aspects of home births. Usually one or two letters concerned a specific physical problem. Sometimes it could be more: March 1982 saw four responses to a feature in the previous issue on a woman’s traumatic and humiliating experience of childbirth in a hospital, plus a letter on Tampax advertising, and one on vaginal warts.^53^

*Spare Rib’*s readership was bigger than any other feminist print community in the country. The magazine’s initial print run was 20,000 in its first year but quickly tailed off to 12,000 (*Cosmopolitan* regularly saw circulation figures of 440,000 in the 1970s).^54^ Despite these relatively low figures, *Spare Rib’*s readership was perhaps double the print run, as women shared copies of the magazine, or read it in libraries or women’s centres.^55^ The magazine aimed from the outset to reach a wide range of women by being nationally available for sale off the shelf, rather than by subscription only, the voluntary distribution networks relied on by other movement publications, or the vigorous but patchy network of feminist bookshops.^56^ Rose Ades, the magazine’s first business manager, recalled how proud she was of *Spare Rib* being sold on the news-stand, including in WH Smith, insisting that such engagement with the market—unique among movement publications—was the ‘only way to reach a wider audience’.^57^ That wider audience became visible in the gamut of emotions, registers and topics of its letters pages, and in the numerous ‘classifieds’ listings from women who depended on *Spare Rib* to find groups near them.^58^

When it came to health, *Spare Rib* offered a political seriousness, and a commitment to anatomical and emotional frankness that was unique on the newsstands. Sue O’Sullivan insisted in the Spare Rib Health Reader of 1987, ‘ … the kind of articles which make up this collection could only have appeared in the numbers and with the consistency they have in a feminist magazine’ and indeed that the ‘themes and considerations’ raised ‘would never find a place in any other magazine, even when the same subject is being covered’.^59^ A backlist of *Spare Rib* issues on health was offered in October 1977, with 62 entries, and occupying two-thirds of the page.^60^ This list detailed the medical content—‘NOT including abortion, campaign news, childbirth or mental health’—of 41 out of the first 62 issues of the magazine, with features covering hysterectomy, PMT, spermicides, thrush remedies, cancer and issues around contraception. Scholars have emphasised the collaborative nature of *Spare Rib*, where readers were called upon to shape and respond to editorial decisions.^61^ The relationship went both ways, and was particularly distinctive in relation to health.^62^*Spare Rib* was pro-active in helping readers, offering information and support where possible.^63^ The collective was attentive to pressing concerns expressed repeatedly, bringing readers, the magazine and health groups together, and in some cases, facilitating the formation of health groups, as in the case of the herpes association that formed after the magazine’s ground-breaking coverage of the disease.^64^*Spare Rib* helped build its community of sufferers beyond the letters pages, by inviting women to write first-person features in articles such as ‘Vaginismus: I Tried to Make Love But I Closed Up Completely’, ‘Abortion: Diary of a Nightmare’, and ‘No such thing as pain: Having your baby in hospital is supposed to be safe and restful … Ruth Wheeler knows otherwise!’.^65^ Where appropriate, the magazine reached outward, or drew inwards, to facilitate the solidarity of bodily experience. A feature on endometriosis was written by Ailsa Irving, a sufferer who told how after writing a letter to the *Guardian* seeking others with the condition she started an endometriosis self-help group and then a newsletter.^66^ The article for *Spare Rib*, called ‘Endometriosis: A Monthly Cycle of Problems’, boldly synthesised description of the physical details of the malady with an unstinting description of the pain of ‘debilitating abdominal cramps’.^67^

*Spare Rib’*s health agenda and coverage was enabled by the way the magazine was run. Launched in 1972 on a budget of £2,000, *Spare Rib* became collective-run in 1974, a move that clarified its critique of the hierarchies of traditional commercial enterprise and patriarchal institutions more broadly. Such ethics shaped its editorial content and publishing practice—particularly in the rejection of mainstream advertising. The magazine was therefore free not only to offer thoroughgoing critiques of commercial body and beauty messaging but to embrace a feminist concept of health, understood as a clash between patriarchal medicine, inscribed on women’s bodies, and women’s own experiences of embodiment and horizon of wellbeing. Returning to Baker’s comments, mainstream women’s magazines, while encouraging intimate disclosures, discouraged a critical or political framing of the body, and created forums in which ‘health is … something which calls for individual hard work, not social solutions’.^68^*Spare Rib*, by contrast, invited the expressions of negativity attached to the isolation, fear and pain of wayward or inscrutable biology. In its pages, women were offered the chance to express themselves against a paradigm that said: ‘[women] can do something about ourselves. We can change our attitude by doing some work on our bodies’.^69^*Spare Rib* readers, tired by the mainstream approach to ailments, seized the chance to write with unvarnished negativity about reality. Cheered by a discussion on migraines with ‘real women about real lives’, hosted by *Spare Rib* writer Eileen Fairweather, a reader shared her frustration with the advice in women’s magazines, ‘that crazy stuff [saying] write diaries and watch films to ease the suffering!!! Have [the people recommending this] ever had a migraine? Any activity is out … ’.^70^

In its total critique of both patriarchal medicine and the spirit of ‘healthism’, *Spare Rib* established a discursive space in which the shame and embarrassment associated with the most ‘unfeminine’ aspects of women’s bodies—their secretions and failings—were accepted, normalised and encouraged. The magazine was a catalyst for collective (positive) empowerment through the provision of information. But it was also, crucially, a negative space, a space in which disempowerment could be explored and physical suffering foregrounded. In describing their bodies in this way, *Spare Rib* readers exercised the unladylike freedom to complain about issues associated with their reproductive organs, and to do so in extreme detail. Although readers wrote about a range of complaints, and the shame and suffering that went with them, from skin problems to hair loss, it was in their epistles about genital misery that *Spare Rib* readers dismantled feminine norms of cryptic, hinted or partial disclosure, and where their anger, pain and complaint felt at their most potent.

The following section examines two types of reader accounts. In the first, the specificity, singularity, and the recoding of experience can itself be read as a political act, bringing out into the open what was not normally codified, and which remained off-limits in women’s magazines.^71^ These letters exuded emotional relief, and were solution-focussed, used by their writers for the chance to tap into a community of shared experience and knowledge, and to participate in a feminist informational economy. A second type of letter brought the health establishment and authorities into view in conjunction with the expression of productive anger, and, in some cases, enabled women to insert their bodies in much grander narratives, using their experience as a way of critiquing feminism, capitalism, and the state.

## Letter Selection: 1972–1988

The ability to control the shape and narrative of these divulgences through writing, without the need to process the immediate feedback and response visible in CR groups, tapped into a key strand of feminist self-expression in print culture.^72^ Susan Wells has observed that the ‘bare taxonomy of anatomical description’ in *Our Bodies Ourselves* fulfilled several political aims, including the countering of ‘anxiety and disgust’ in relation to women’s bodies, and in making visible the ways in which female bodies were ‘territories … inscribed with … oppression’.^73^*Spare Rib* readers moved to write to the magazine about bodily misery did not always make these connections explicitly, but seemed to seek the ‘sisterly’ possibilities of sharing such experience, and elucidating its horrors without trying to impose tonal restraint or silver linings.^74^

Frank self-narration provided a basic launchpad for entering the informational paradigm underpinning the women’s health movement. Having detailed the problem, readers then asked for help, a translation of sorts of the agony aunt model—but with fellow readers and the magazine as the provider of comfort and information.^75^ Since the 1920s, agony columns had been a touchstone of the intimate, dependent relationship between readers and women’s magazines in Britain and the US, providing help and reassurances unavailable elsewhere, and—crucially—bringing intimate disclosures into the realm of ‘the sayable’.^76^*Spare Rib* had a short-lived problem column with Anna Raeburn, and its decision to discontinue it in 1974 may have been to do with its provision of other spaces for reader response on one hand and expert provision of information (for instance in lengthy pieces of health reportage) on the other, or, as the magazine evolved, unease at featuring a hallmark of traditional women’s magazines.^77^ Certainly, *Spare Rib’*s letters page offered a strong, grass-roots alternative to an agony page presided over by a knowing ‘aunt’, and it was solidarity in suffering that characterised these letters. Those experiencing bodily anguish addressed their audience in ways directly linked to their own radical disclosures. A woman suffering from vaginal warts began her letter: ‘Can any of your readers help me?’ She then told how ‘Just over a year ago I had a wart—just inside the lips of my vagina … ’.^78^ A reader with an ‘incorrect womb’ told how she had been ‘offered an operation to “correct” a retroverted uterus, mainly because for years I have suffered from fairly frequent stabbing pains during intercourse’.^79^ The procedure was elucidated in forensic detail, involving: ‘lifting the uterus to a normal position and securing it with permanent sutures … leaving what the doctors describe as ‘a small scar below the bikini line’. The woman’s quest for information about this condition and procedure had been unsuccessful, and, having laid out her experience, she now sought ‘the experiences and views’ of ‘any other women who have had this operation’. A reader from Lancashire wondered if the use of the cap caused vaginal wall abrasion; she experienced secretions, inflammation, and itchiness on removal, with ‘pieces of skin discharged … I don’t seem able to find the cause’.^80^ The letter assumed a community caring enough to take an interest in wholly personal dilemmas and happy to provide information on that basis, while demonstrating ease with describing the most intimate forms of ill health. Bodily disclosures made, the reader concluded with the familiar request for help: ‘I would be grateful for any help as I do not want to be in the position of having to rely on the rhythm method or condoms as a method of birth control’. Explicit accounts of vaginal wall abrasion or the experience of having a ‘retroverted’ uterus corrected reveal how readers’ engagement with a broader feminist community took shape through the force of ‘the bare taxonomy of anatomical description’.

A number of anatomically grounded letters reached beyond the request for communal input, packing a political punch through their emotional register, and particularly through negativity or anger and the refusal to see a silver lining. Describing a contraceptive nightmare in 1973, Suki Pryce, a reader from Somerset, wrote in response to an article on the IUD. In it she set out to ‘tell you about my crummy experience with the coil’. ^81^ She offered an account of the cramps, bleeding and pain during sex that followed insertion of the coil, and identified the site of pain as her uterus and womb, which, she said, had begun to push the coil out. A clear chronology of detailed suffering and medical mismanagement alchemised the point and politics of the letter. After relating how the coil was pushed out, the letter continued with the story of an infection. Pryce had to be hospitalised, and on release suffered discharge and more flare-ups of the infection. Having detailed what happened Ms Pryce resolved her letter with a refusal to forgive or look on the bright side, turning to the ‘intimate public’ of fellow readers galvanised be their own experiences of discomfort and anger. She had become, she said, a ‘sexual cripple’, hoped she had not been ‘messed up for good’ by the coil, and invited others to come forward. Other letters followed a similar logic, not all as eloquent in their fury. In a letter headed ‘Down at the special [STI] clinic’, a reader told of suffering ‘a lot of humiliation and patronising’ in being diagnosed with non-specific-uthretitis, a form of chlamydia.^82^ Her letter asserted its belonging to the feminist milieu with a furious question about what she paid her National Insurance for and the sign off ‘In anger, Isobel, Glasgow’.

The seriousness with which the depressive aspects of poor health were treated formed a crucial part of the magazine’s approach to embodiment. An article entitled ‘Pelvic Inflammatory Disease’ in which sufferer-authors told how ‘over the years they have discovered a large number of women struggling to cope with this draining and painful gynaecological ailment’, stressed the role of the *Spare Rib* community in countering the ‘psychological stress and depression this disease can bring’.^83^ Readers took up the invitation to describe the psychological discomfort that accompanied their maladies. The vaginal warts sufferer began to be ‘be irritable and depressed’.^84^ The psychological and physical effects of the pharmaceutically engineered hormones of the Pill garnered particularly detailed attention. In one typical account, a reader told of having

been on the Pill for seven years. In the beginning it was to help with my painful periods. I stared with Ovulen-5- but it made me depressed. I changed to Minilyn, but my periods started to get painful again. In September ’75 I changed to Eugyon. I became less and less interested in sex. Even repulsed by it …^85^

Another reader, detailing her traumatic experience with the birth control pill, sought information and solidarity while also including the negative palate of feelings the experience created. Having been prescribed the birth control pill Norinyl five years previously, she developed Stein-Levanthal syndrome, ‘which meant that the ovaries were covered with cysts and the periods stopped’. The reader told how she was ‘most distressed by the fact that my face was very swollen’. Following an operation on her ovaries, her periods returned and her surgeon declared her better, though

my face has never returned to normal. I feel more and more isolated by this illness, because I have so little information about it, because I have never met anyone else who has had it, and because this face does not reflect my own identity, I feel trapped behind it. If anyone has any knowledge of this illness I would love to hear from them.^86^

While mainstream women’s magazines fielded a range of accounts detailing health complaints, including those that were upsetting or caused despair, *Spare Rib’*s explicitly feminist commitment to politicising the personal opened up the emotional horizon for readers in a different way, freeing up a range of feelings that could, in its pages alone, be linked to the oppressive and cumulative effects of patriarchy on women’s bodies and minds.

## From Pain to Politics

*Spare Rib* fostered a correspondence environment in which readers could share the details of the secretions and infections of their reproductive organs with full transparency. In presenting such material in a space both public and supportive, women made the first transition from the individuated to a shared experience of embodiment. For some, however, body letters enabled a further step, reaching outwards towards grander narratives which firmly positioned their own private experiences in the politics of women’s liberation.

Reproductive issues inspired a particularly robust linkage of bodily experience with political analysis, as readers detailed a range of interconnected and seemingly unresolvable problems. ‘Six months ago I became pregnant while using the cap’, wrote Sarah Kirkham in November 1975.^87^ She was lucky to be ‘granted the favour’ of an NHS abortion after a great deal of humiliation and had ‘since been told that, in fact, owing to the position of my uterus, the cap was unsuitable’—she also had forebodings about the progesterone-only pill. These experiences, and the suggestion that her body was not amenable to contraception, had led her to a personal and political interest in menstrual extraction or interception which, she felt, ‘could play a very important role in the struggle for control of our own bodies’. A similar pattern was at play in the account of Julie Carter in June 1981. ‘I am trying to contact other women who are suffering from, or have suffered from vaginal and/or peri-anal pruritis’, she wrote.

I have had to contend with this demoralising, painful and irritating illness for about seven years. The specialist I am currently seeing says that he sees at least two female patients per week suffering from pruritis. I want to contact other women in the same situation. Then we would have the power to pressurise the medical authorities.^88^

This writer positioned the medical establishment in two ways: as a neutral entity helping her, (‘the specialist I am currently seeing’) and as a sexist and neglectful body, requiring the political ‘pressure’ women could create collectively, facilitated by, or at least imagined, through *Spare Rib*.

Jacqueline Wright outlined her experience of ‘prolapse repair’, of being pressured to have a child asap to make way for a hysterectomy, and of being treated brusquely and patronised, her questions unanswered by the medical staff concerned.^89^ After a full account of what happened to her, she wrote: ‘I don’t see why women should let men just hack their bodies around and not feel able to have a sit-down and talk about it’. Others went further, emboldened by *Spare Rib’*s editorial coverage. In response to an article about hysterectomy—‘Last week I was rushed into hospital with excessive menstrual bleeding and appalling stomach pains … ’ wrote Jo Evans, who was then diagnosed with a cyst.

Armed with your article, Our Bodies, Ourselves and discussions with a few medical friends, I will return to the hospital and tell the consultant how outrageous his suggestion is. He should stop whipping the wombs out of the women of Bristol and resign so that more women can rise to the top of the gynaecological profession….^90^

After seeing an article on the coil in *Spare Rib*, another reader was prompted to get hers taken out. She made the appointment, and told how when the blue string started to emerge on its own she went to the Royal Free hospital for emergency removal and was ridiculed her for having had an abortion at 17. The doctor ‘made me feel like a rabbit, not a woman in tune with or in charge of my own body. The sooner women’s self-help groups catch on, and women take group responsibility and remove the medical alienation of women’s bodies the better’.^91^ Reader experience was also sought for documentation to help change public awareness. Caroline Thorpe, Lisa Wood ‘and their husbands’ wrote detailing a terrible experience with the Copper 7 coil. ‘I had to have both fallopian tubes and one ovary removed’ they wrote, ‘as they were irreparably damaged and septic … ’.^92^ The letter pivoted to a call to action, sharing findings that up to 20,000 women have ‘tubes’ problems and are infertile with a possible link to the Copper 7. This, they concluded, meant there ‘may be many more [and] We’d like to hear from readers with similar experiences’. Such letters showed how Spare Rib readers could interpret bodily experience: as an event to be shared in detail in a public forum, deployed to find and forge unity with other sufferers, an opportunity to collect and collate data, and from there, a spur to group action.

## Letters 1989–1993

In its first 15 years *Spare Rib’*s correspondence pages offered a space in which readers could deploy novel modes of language that broke conventions of euphemism and embarrassment when it came to their bodies, and specifically their vaginal and reproductive health. Throughout the 1980s, in step with regular features and news stories, readers continued to write about ill-health, reproductive issues and sexist handling by the medical establishment but by 1988 the letters section had become more focussed on questions of race, internationalism and intersectional oppressions. Experiences of physical malaise, however much a starting point for collective action, were nonetheless rooted in the individuated world of the body, and the kind of letters considered above faded from view in the final years of the magazine. Readers noted and commented on the shift from readers’ physical worlds outwards to an internationalist terrain that often seemed to them to privilege certain vectors of oppression over gender, and particularly women’s experience in Britain. Indeed in July 1989, *Spare Rib* dropped its tag, ‘A Women’s Liberation Magazine’, which it had adopted in 1976. The choice by *Spare Rib’*s now all-black collective to return to a blank banner reflected the divisiveness of the word ‘women’ by the late 1980s, which could be associated with an elitist white version of womanhood and feminism.^93^ From 1990 onwards, readers argued over the magazine’s decision to foreground international concerns and race over, as some felt, class, sexuality and even gender. While some applauded the focus on intersectionality and global politics, a number of long-time subscribers wrote in explaining why they were giving up their subscription. In their view, the unifying vision of womanhood and inclusive attitude towards the readership’s experience had given way to a harsh and exclusionary intellectualism. Reader Julie Thompson wrote in November 1990 that *Spare Rib* had ‘lost its original purpose … *Spare Rib* has moved from Active Grassroots to Superstars, Political Theory and International Relations’.^94^ She called for the reinstatement of Consciousness Raising listings those of other women’s groups, including health groups. In February 1990, a Manchester feminist in search of ‘a sisterhood’ accused *Spare Rib* of being not a periodical for feminists but rather ‘A PERIODICAL FOR THE GIRLFRIENDS AND WIVES OF RADICAL MEN’.^95^

Anger still animated the letters pages, but it was increasingly directed towards the movement and to *Spare Rib*, and letters like the above were positioned as firing shots for lengthy disputes between readers. The fruitful quest for solidarity that had emboldened women to write about their experiences of genital ill-health was replaced by intra-reader antagonism. Between these disputes concerning *Spare Rib’*s contents and the magazine’s increasingly internationalist priorities, the more open-ended sense of exchange previously fostered between readers in the 1970s and 1980s was lost.

Those letters that did concern women’s bodies zoomed out from the struggles with pain, disease and discomfort with reproductive organs. In July 1989, for instance, two letters concerned women’s bodies, but both were written in response to an article by Pat Spallone, a bioethicist.^96^ They debated the politics of genetic engineering, IVF and the role of women in science, and were followed with a reply from Spallone responding to their critiques. In May 1990, the health-related correspondence came from the Women’s Global Network for Reproductive Rights in the Netherlands on International Day of Action for Women’s Health, while five out of six letters in the June 1990 issue concerned debates over embryo research, weighing up harms of capitalism versus the liberatory promise of research. No further letters appeared concerning women’s individual experiences of ill health.

*Spare Rib’*s shifting priorities reflected changes in the politics of the women’s liberation movement as it moved from a flexible personal–political model to a more contested emphasis on the politics of intersectionality and imperialism. In *Spare Rib’*s terms, this included reckoning with who had been included in and who excluded from the sisterhood—including the sisterhood of health misery—in the 1970s and early 1980s, prompting efforts to shift the epistemology of health in *Spare Rib* and the wider women’s movement towards a less apparently white-focussed concern.^97^ These changes took place against a backdrop of new institutional arrangements in women’s health, which had become more systematised and brought into line with state provision, denuding organisations of their early focus on group sharing of experience. The health movement had expanded and diversified in the 1980s, providing a vast web of forums for medical advice and information. By the late 1980s there were attempts to consolidate its almost comic complexity. The first issue of the Women’s Health and Reproductive Rights Information Centre Newsletter in June 1988 told how the ‘WHRRIC (pronounced with a silent “h” please!) combines the Women’s Health Information Centre (WHICH) and the Women’s Reproductive Rights Information Centre (WRRIC).^98^ WHICH dealt with wider women’s health issues such as abnormal smears, breast cancer, PMT and thrush, whereas WRRIC dealt with reproductive health issues such as abortion, contraception, infertility and reproductive technology’. Looking back on the growth in interest in women’s health ‘over the last 15 years’, the Women’s Health Network explained in a flyer how it had been forged from an influential Better Health for Women conference in 1986, had hosted a successful national conference in Liverpool in 1989, and was now based within the National Community Health Resource (NCHR) in London, funded by grants from the Equal Opportunity Commission, War on Want and the World Health Organization. ^99^ Its goals lacked feminist inflection, concentrating on policy, to ‘provide info on women’s health projects etc; inform regional policy makers; develop links between groups and individuals and develop local and regional networks’.^100^ By 1990, such organisations were part of an extensive network of sites in which women could voice and seek treatment for bodily complaints. The loss of health-related vitality in *Spare Rib’*s readers’ forum coincided with their emergence. Bodily misfortune had become atomized and specialized in medical provision and a growing array of services, rather than remaining a spur to collective revolt.

This article has focussed on *Spare Rib’*s contribution to the women’s health movement. It has drawn out the magazine’s epistolary and editorial distinctiveness in relation to the feminist ecology of health groups and other women’s magazines, and posited the magazine as a key source for understanding how bodily experience drew a range of women into a direct appreciation of feminist politics in 1970s and 1980s Britain. Through its epistemological commitments to information provision, radical frankness and the authority of experience, along with an emotional agenda encouraging the expression of negative or angry sentiment, *Spare Rib* offered a distinctive environment for intimate disclosures. It was therefore uniquely positioned in its correspondence pages to enable a range of non-activist women to discover for themselves the bridge between individual experience and the politics of feminism. In its final years, new political commitments threw the very category of woman into doubt, and with it, complaints rooted in individual women’s bodies. *Spare Rib* ultimately lost the open-ended sense of exchange between readers, and between readers and the magazine that had previously made it Britain’s key forum encouraging the articulation of political selfhood rooted firmly in the taxonomies of bodily disorder and pain.

## Footnotes

1 Adrienne Rich, *Of Woman Born: Motherhood as Experience and Institutio*n (London: Virago, 1977).

2 See, for example, Barbara Ehrenreich and Deirdre English, *Complaints and Disorders: The Sexual Politics of Sickness* (London: Compendium, 1974).

3 Lynne Segal, *Why Feminism? Gender, Psychology, Politics* (Cambridge: Polity, 1999); Sarah Crook, ‘Reproductive Rebellions in Britain and the Republic of Ireland: Contemporary and Past Abortion Activism and Alternative Sites of Care’, *Feminist Encounters: A Journal of Critical Studies in Culture and Politics*, 2018, 2; Kate Mahoney, ‘Finding our own solutions': the women's movement and mental health activism in late twentieth-century England’ (PhD thesis, University of Warwick, 2017).

4 Laurel Foster, ‘Printing Liberation’, in Laurel Foster and Sue Bruley, eds, *British Culture and Society in the 1970s: The Lost Decade* (Newcastle: Cambridge Scholars: 2010), 100.

5 Sue Bruley, ‘Consciousness-Raising in Clapham; Women's Liberation as “Lived Experience” in South London in the 1970s’, *Women’s History Review*, 2013, 22, 717–738; Caroline Smith, ‘Promotion of Medical and Biological Group’, Women’s Liberation Workshop Newssheet, No. 26 (4 April 1971), 2 (Feminist Library); Highbury and Stoke Newington Women’s Group, ‘Free Pregnancy Testing and Information’, Women’s Liberation Workshop Newsletter, No. 114 (31 December 1972), 2 (Feminist Library).

6 Katherine Holden, ‘Nature Takes no Notice of Morality, Singleness and Married Love in Interwar Britain’, *Women’s History Review*, 2007, 11, 481–504; Margaret Jackson, *The Real Facts of Life: Feminism And The Politics Of Sexuality c. 1850-1940* (Milton Park: Taylor and Francis, 1994); Roy Porter and Lesley Hall, *The Facts of Life: The Creation of Sexual Knowledge in Britain,*1650–1950 (New Haven: Yale University Press, 1995), 248.

7 Lesley Hall, *Hidden Anxieties: Male Sexuality*, 1900-1950 (Cambridge: Polity, 1991).

8 Margaretta Jolly, *In Love and Struggle: Letters in Contemporary Feminism* (New York: Columbia University Press, 2008).

9 Barbara Green, ‘The Feel of the Feminist Network: Votes for Women after The Suffragette’, *Women: A Cultural Review*, 2017, 27, 359–77.

10 Melanie Waters, ‘Yours in Struggle’: Bad Feelings and Revolutionary Politics in Spare Rib’, *Women: A Cultural Review*, 2017, 27, 446–65, 456.

11
*Ibid*., 451.

12 Lesley Doyal, ‘Women, Health and the Sexual Division of Labour: A Case Study of the Women’s Health Movement in Britain’, *Critical Social Policy*, 1983, 3, 21–32, 22.

13 Bruley, ‘Consciousness Raising in Clapham’; Sheila Rowbotham, *Woman’s Consciousness, Man’s World* (Harmondsworth: Penguin, 1973); Eve Setch, ‘The Women’s Liberation Movement in Britain 1969–79’, *Twentieth Century British History*, 2002, 13, 171–190. Health collectives and informational services, both local and national, proliferated; the National Joint Committee of Working Women’s Organisations on Women’s Health in 1977 released a report covering no fewer than 17 areas from health care finances to cancer prevention. There were attempts to simplify disparate informational sources: one bold example was the Women’s Health Information Centre newsletter founded by Lesley Doyal, Nancy Worcester and Gill Yudkin (First issue Autumn 1983). FAS, DM2123/5/8.

14 Politics of Health Group, ‘Cuts in the NHS’, (1980) London, and ibid., (1982), ‘Going Private’, London.

15 Doyal, ‘Sexual Division of Labour’, 24.

16 Feminist Archives South (FAS) and North (FAN) offer insight into the range of health areas covered by groups and newsletters: see FAS, Health Topic Boxes 1-16, DM2123/2; FAN, FAN/EOC/06.

17 Attaining full cooperation with the NHS was a patchy and uneven process. The author of the Weston Community Health Council Annual Report of 1978 noted that considerable ‘enthusiasm’ for a local Well Woman clinic failed to provide ‘sufficient impetus to the District Management Team to get a clinic started under the NHS’, FAS, DM2123/2/HCM 1, Manila folder.

18 Islington Community Health Council (CHC) News, January 1978, FAS, DM2123/2/HCM 1, Manila folder.

19 Letter from Lynn Roberts of Islington Community Health Council to Mr Dunning, 17 August 1981, FAS, DM2123/2/HCM 1, Manila folder.

20 ‘Well Women In Manchester? First Report: 22 November 1979’, DM2123/2/HCM 1, Manila folder.

21 Feminist Women’s Health Centre Presents the Participatory Clinic (LA/Santa Ana, California), DM2123/2/HCM 1, Manila folder.

22 Doyal, ‘Sexual Division of Labour’, 22.

23 Kathy Davis, *The Making of Our Bodies, Ourselves: How Feminism Travels Across Borders* (Raleigh: Duke University Press, 2007).

24 Ibid., 2.

25 25 See, for example, Rakusen and Philips on importance of state to British women’s health activism, 7–10; in American context, Ehrenreich*, Complaints and Disorders*.

26 On the US women’s health movement’s engagement with the medical profession and health policy-makers, see Jennifer Nelson, *More Than Medicine: A History of the Feminist Women's Health Movement* (New York: NYU Press, 2015); Wendy Kline, *Bodies of Knowledge: Sexuality, Reproduction, and Women's Health in the Second Wave* (Chicago: University of Chicago Press, 2010); Sandra Morgen, *Into Our Own Hands: The Women's Health Movement in the United States, 1969-1990* (Rutgers: Rutgers University Press, 2002).

27 Adrian Bingham, *Family Newspapers: Sex, Private Life, and the British popular press 1918-1978* (Oxford: Oxford University Press, 2009), 49.

28 Porter and Hall, *The Facts of Life*, 147.

29 Rosemary Pringle, *Sex and Medicine: Gender, Power, and Authority in the Medical Profession* (Cambridge: Cambridge University Press, 1998).

30 Hera Cook, *The Long Sexual Revolution: English Women, Sex, and Contraception, 1800-1975* (Oxford: Oxford University Press, 2004), 145.

31 Holden, ‘Nature Takes No Notice of Morality’. Marie Stopes’s *Married Love* in 1918 offered detailed advice on the minutiae of female sexual stimulation. An ardent eugenicist keen on birth control in order to restrain working class over-reproduction, Stopes only dispensed advice to married women, and single women who ran into bodily difficulty because of sex were treated harshly.

32 Wendy Savage, *A Savage Enquiry: Who Controls Childbirth?* (London: Virago, 1986), 59; on women in gynaecology, see Pringle, *Sex and Medicine*, 43, and Mary Ann Elston, ‘Women Doctors in the British Health Services: A Sociological Study of Their Careers and Opportunities’ (PhD thesis, University of Leeds, 1986).

33 Nancy Mackeith, *The New Women’s Health Handbook* (London: Virago: 1978), 5.

34 Jill Rakusen and Angela Phillips, eds, *The New Our Bodies Ourselves* (London: Penguin, 1989), 19.

35 See Hera Cook on interwar prescriptions, *The Long Sexual Revolution*, 143–165.

36 Daisy Payling, ‘Experience and Expertise in 1960s Women’s Magazines’, Expertise of Experience symposium, 27 April 2019, London Southbank University.

37 Kate Muir, ‘Dark Ages’, *The Times*, 22 April 2006, p. 9.

38
*Nova*, Freshette July 1972; *Nova*, Wella advert January 1975, 6; Ibid., British Airways advert, 7; Ibid., Crème de Menthe, 25.

39 Susie Orbach, *Fat is a Feminist Issue* (London: Hamlyn, 1979), Joan Jacobs Brumberg, *Fasting Girls: The Emergence of Anorexia Nervosa as a Modern Disease* (Cambridge, MA: Harvard, 1988); for a critical overview of the literature on increasing rates of anorexia in the 1970s: Sonja Van’t Hof, ‘The Rise and Fall of A Fact: The Increase in Anorexia Nervosa’, *Sociology of Health and Illness*, 1996, 18, 581–608.

40 The Business of Women’s Words, interview with author, Cheltenham, 2019.

41 The numbers of women paying to attend fitness classes and gyms rose from 2 per cent in 1977 to 17 per cent in 1992, the gym business growing by another 58 per cent to £1bn by 1998. Ina Zweiniger-Bargielowska, *Women in Twentieth-Century Britain: Social, Cultural and Political Change* (London: Routledge, 2001), 190. Katrina Moseley (2019) has tracked the meteoric rise of the slimming industry in Britain, which by 1983 was worth an estimated hundred million pounds. ‘Slimming one’s way to a better self? Weight Loss Clubs and Women in Britain, 1960—1990’, unpublished article. Meanwhile, the sense that women’s bodies could be rendered legible through biotechnological innovation helped drive the success of the commercial diagnostics industry in Britain in the 1970s and 1980s; by the 1990s, pregnancy tests had become its most lucrative sector. Soraya de Chadarevian, ‘The Making of an Entrepreneurial Science: Biotechnology in Britain, 1975–1995’, *Isis: A Journal of the History of Science*, 2011, 102, 601–33. New methods for sanitising women’s bodies emerged in the 1970s too, with Playtex introducing its first deodorant scented tampon in the US in 1971.

42 ‘Hairy Story’, January 1978, 6–8.

43 Outside of *Spare Rib*, harrowing first person accounts tended to relate to single issues. For instance: *Women’s Health – AIMS Journal* (Association for Improvements of Maternity Services – founded 1960), ‘I was very angry- I was having a baby, not a miscarriage!’ 10 (3), Winter 1998/9, 4–6. The title page of a draft of a health education council publication on menopause (July 1984) had numerous quotations—‘Indescribable hell: that’s what the menopause was like for me’ and ‘We don’t talk about it -not in our community. We just try to hide it!’—but no in-depth experience. FAS DM2123/2/HCM2, brown folder.

44 Beyond CR, it was a vital forum for advertising group activity, with up to 10 pages of notices of activist news and meetings. It ran feminist business adverts on its inside cover, in dedicated adverts pages, and in the Classifieds.

45 Women’s health groups were also committed to an agenda of reaching the widest possible number of women whereas, although *Spare Rib* embraced the marketplace in order to a wide range of readers nationally, its priorities were more in step with the women’s liberation than ‘women’. The Swindon Women’s Health Group Newsletter informed members of its intent to attract ‘hordes’ of women by moving to bigger venue, though the move was too pricey and so the idea was abandoned. Its agenda was inclusive rather than political: offering fortnightly meetings with speakers to discuss topics including alternative medicine, natural childbirth, homeopathy, diet, well-being, bereavement. FAS, DM2123/2/HCM 1, Manila Folder.

46 Well Woman Scoping Report, 1979.

47
*Spare Rib* was itself scathing about *Cosmopolitan*’s consumerism and individualism, and especially its ‘methodically trite’ coverage of women’s issues, while as Laurel Foster suggests, the magazine was at times distinctly non-feminist. ‘Printing Liberation’, 101.

48 Hera Cook, ‘Nova 1965–1970: Love, Masculinity and Feminism, but Not as We Know It’, in A. Harris and T. W. Jones, eds, *Love and Romance in Britain, 1918–1970. Genders and Sexualities* (London: Palgrave, 2015), 225–244.

49
*Nova*, January 1975, 8.

50
*Cosmopolitan*, February 1975, 116.

51
*Cosmopolitan*, January 1984, 144.

52
*Company*, January 1989, 5.

53
*Spare Rib*, March 1982, 3.

54 Janice Winship, *Inside Women’s Magazines* (London: Rivers Oram, 1987), 166.

55 ‘Women’s history month: Spare Rib’, Women’s History Network, 22 March 2011, https://womenshistorynetwork.org/womens-history-month-spare-rib/.

56 Lucy Delap, ‘Feminist Bookshops, Reading Cultures and the Women’s Liberation Movement in Great Britain, c. 1974–2000’, *History Workshop Journal*, 2016, 81, 171–196.

57 Interview with author, The Business of Women’s Words, The British Library, October 2018, Track 2.

58 Heterogeneous use was made of the groups search listings, for example, ‘Any schoolgirls in the Reading area interested in starting a group?’; ‘Call to form older women’s group in Manchester area’. Both *Spare Rib*, June 1979, 30.

59 Sue O’Sullivan, *Women’s Health:* A *Spare Rib Reader* (London: Pandora, 1988), 2.

60
*Ibid*., 17.

61 Lucy Delap and Zoe Strimpel, ‘*Spare Rib* and the Print culture of Women’s Liberation’, in Laurel Forster, ed., *Women, Periodicals and Print Culture in Contemporary Britain* (Edinburgh: EUP, 2020).

62 Susan Ardil, ‘Health and Spare Rib’, British Library, https://www.bl.uk/spare-rib/articles/health-and-spare-rib.

63 A commitment to helping readers, even when their letters could not be printed, can be seen in the Feminist Archive South *Spare Rib* cache.

64 Ardil, ‘Health and Spare Rib’.

65
*Spare Rib*, ‘Vaginismus’, November 1974, 34, Ibid., ‘Abortion’, June 1980, 6, Ibid., ‘No Such Thing as Pain’, January 1982, 51.

66
*Spare Rib*, June 1983, 37.

67
*Ibid*., 36–40.

68 Rosalind Coward, *Female Desire* (London: Paladin, 1984), 25.

69
*Ibid*., 22.

70
*Spare Rib*, January 1980, 4.

71 Angela McRobbie, *Feminism and Youth Culture: From Jackie to Just Seventeen* (Basingstoke: Macmillan, 1991); Michaela Frischherz, ‘Cosmo Complaints: Reparative Reading and the Possibility of Pleasure in Cosmopolitan magazine’, *Sexualities*, 2017, 21, 552–68.

72 Laurel Forster, ‘Spreading the Word: Feminist Print Cultures and the Women's Liberation Movement’, *Women’s History Review*, 2016, 25, 812–31.

73 Susan Wells, ‘Our Bodies Ourselves: Reading the Written Body’, *Signs*, 2008, 33, 697–723, 703.

74
*Ibid*.

75 Anna Raeburn’s sex problems page disappeared in 1974. *Spare Rib* did not have an agony aunt or any regular figure to address such questions.

76 Angela Phillips, ‘Advice columnists’, in Bob Franklin, ed., *Pulling Newspapers Apart: Analysing Print Journalism* (London: Routledge, 2008), 102–112, 103; Adrian Bingham, ‘Newspaper Problem Pages and British Sexual Culture Since 1918’, *Media History*, 2012, 18, 51–63.

77 The flux in *Spare Rib*’s identity and direction in its first years as it tried to plot a course independent of the commercial lynchpins of mainstream women’s magazines spanned advertising, content and style. Joanne Hollows, ‘Spare Rib, Second-Wave Feminism and the Politics of Consumption’, *Feminist Media Studies*, 2013, 13, 268–87, 272–73.

78
*Spare Rib*, March 1982, 8.

79
*Ibid*., September 1980, 5.

80
*Ibid*., July 1981, 5.

81
*Ibid*., December 1973, 4.

82
*Ibid*., November 1981, 4.

83
*Ibid*, December 1982, 18–19.

84
*Ibid*., March 1982, 3.

85
*Ibid*., Nov 1977, 9.

86 Susan Goulden, 12 Ossulton Way, London N2.

87
*Spare Rib*, November 1975, 4.

88
*Ibid*., June 1981, 4.

89
*Ibid*., October 1977, 5.

90
*Ibid*., February 1982, 4.

91
*Ibid*., June 1977, 4.

92
*Ibid*., March 1980, 5.

93 Nathalie Thomlinson, *Race, Ethnicity and the Women's Movement in England*, 1968–1993 (Basingstoke: Palgrave). *Spare Rib*’s banners ran ‘women’s news magazine’ from 1972, alternating with ‘women’s magazine’ in 1973 before in July 1976 tagging itself as a Women’s Liberation Magazine.

94
*Spare Rib*, November 1990, 5.

95
*Ibid*., 5.

96
*Spare Rib*, July 1989, 4.

97 Ardill, ‘Health and Spare Rib’. For analysis of *Spare Rib* and diversity in the 1980s, see Natalie Thomlinson, ‘(2017) Sisterhood is Plain Sailing?’ Multi-racial Feminist Collectives in 1980s Britain’, in K. Schulz, ed., *The Women's Liberation Movement: Impacts and Outcomes* (New York: Berghahn, 2019) and Thomlinson, ‘Race, Ethnicity and the Woman’s Movement’.

98 Women’s Health and Reproductive Rights Information Centre, No. 1. June 1988, 1.

99 Festival of Women’s Health and Wellbeing (6–7 April 1985); first Women and Health Forum, September 1988.

100 Women’s Health and Reproductive Rights Information Centre, No. 1. June 1988, 1.

## Notes

Zoe Strimpel completed her PhD at the University in Sussex in 2017. Her monograph based on her doctoral work was published in March 2020 (Bloomsbury Academic) and is entitled *Seeking Love in Modern Britain: Gender, Dating and the Rise of ‘the Single’*. She is currently a British Academy-funded postdoctoral fellow at Warwick researching breakups and divorce in late twentieth century Britain.

